# 

**Published:** 1903-03-15

**Authors:** 


### CONTENTS.

Annealing Gold Foil, - - *By J. R. Callahan, D.D.S.* 109

X-ray Vision, or Induced Supernormal Sight, *By J. A. Smalley, D.D.S., M.D.* 115

Dental Legislation, - - - *By James A. McGeough* 127

Compressed Air in Dentistry, *By S. E. Knowles, M. D., D.D.S.* 135

Winning the Confidence of Children, *By Evan P. Wentworth, D.M.D.* 143

Root-canal Filling, - *By B. F. Arrington, D.D.S.* 148

New Local Anesthetic, - - - - - - - 151

Editorial, - - - - - - - - - - 153

Announcements, - - - - - - - - 156

Bibliography, - - - - - - - - 157

Indents, - - - - - - - - - - 159

